# Review of visual odometry: types, approaches, challenges, and applications

**DOI:** 10.1186/s40064-016-3573-7

**Published:** 2016-10-28

**Authors:** Mohammad O. A. Aqel, Mohammad H. Marhaban, M. Iqbal Saripan, Napsiah Bt. Ismail

**Affiliations:** 1 Department of Engineering, Faculty of Engineering and Information Technology, Al-Azhar University-Gaza, Gaza, Palestine; 2 Department of Electrical and Electronic Engineering, Faculty of Engineering, Universiti Putra Malaysia, 43400 Serdang, Selangor Malaysia; 3 Department of Computer and Communication Engineering, Faculty of Engineering, Universiti Putra Malaysia, 43400 Serdang, Selangor Malaysia; 4 Department of Mechanical and Manufacturing Engineering, Faculty of Engineering, Universiti Putra Malaysia, 43400 Serdang, Selangor Malaysia

**Keywords:** Visual odometry, Localization sensors, Image stream, Global positioning system, Inertial navigation system

## Abstract

Accurate localization of a vehicle is a fundamental challenge and one of the most important tasks of mobile robots. For autonomous navigation, motion tracking, and obstacle detection and avoidance, a robot must maintain knowledge of its position over time. Vision-based odometry is a robust technique utilized for this purpose. It allows a vehicle to localize itself robustly by using only a stream of images captured by a camera attached to the vehicle. This paper presents a review of state-of-the-art visual odometry (VO) and its types, approaches, applications, and challenges. VO is compared with the most common localization sensors and techniques, such as inertial navigation systems, global positioning systems, and laser sensors. Several areas for future research are also highlighted.

## Background

Accurate localization of a vehicle is a fundamental challenge in mobile robot applications. A robot must maintain knowledge of its position over time to achieve autonomous navigation. Therefore, various sensors, techniques, and systems for mobile robot positioning, such as wheel odometry, laser/ultrasonic odometry, global position system (GPS), global navigation satellite system (GNSS), inertial navigation system (INS), and visual odometry (VO), have been developed by researchers and engineers. However, each technique has its own weaknesses. Although wheel odometry is the simplest technique available for position estimation, it suffers from position drift due to wheel slippage (Fernandez and Price 2004). INS is highly prone to accumulating drift, and a highly precise INS is expensive and an unviable solution for commercial purposes. Although GPS is the most common solution to localization as it can provide absolute position without error accumulation, it is only effective in places with a clear view of the sky. Moreover, it cannot be used indoors and in confined spaces (Gonzalez et al. 2012). The commercial GPS estimates position with errors in the order of meters. This error is considered too large for precise applications that require accuracy in centimeters, such as autonomous parking. Differential GPS and real time kinematic GPS can provide position with centimeter accuracy, but these techniques are expensive.

**Fig. 1:**
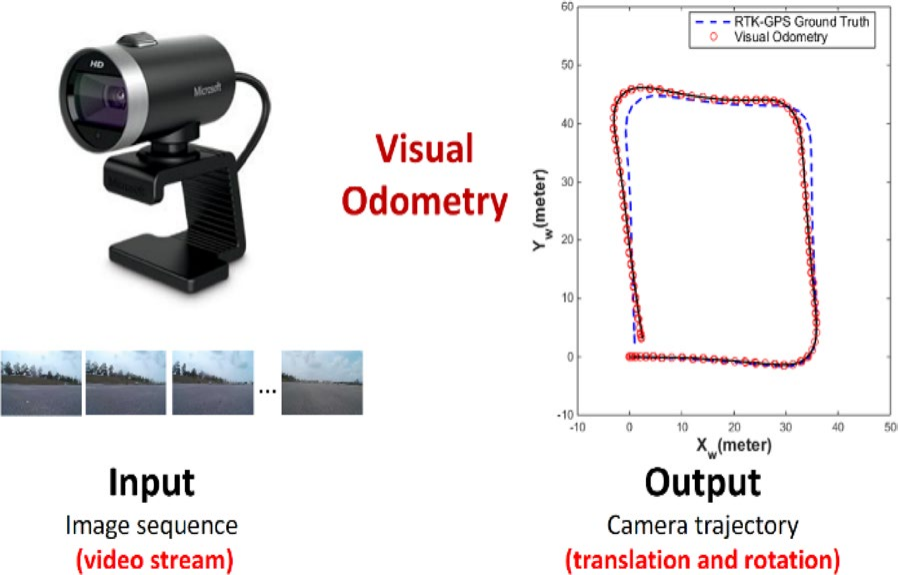
Visual odometry [Aqel et al. 2016]

The term “odometry” originated from the two Greek words *hodos* (meaning “journey” or “travel”) and *metron* (meaning “measure”) (Fernandez and Price 2004). This derivation is related to the estimation of the change in a robot’s pose (translation and orientation) over time. Mobile robots use data from motion sensors to estimate their position relative to their initial location; this process is called odometry. VO is a technique (shown in Fig. 1) used to localize a robot by using only a stream of images acquired from a single or multiple cameras attached to the robot (Scaramuzza and Fraundorfer 2011). The images contain a sufficient amount of meaningful information (color, texture, shape, etc.) to estimate the movement of a camera in a static environment (Rone and Ben-Tzvi 2013).

The article is organized as follows. The next section presents the six most common sensors and technologies utilized for localization in robotic applications and compares their advantages and disadvantages. “VO” section provides a detailed discussion on VO and its types, approaches, applications, and challenges. Prior related works are presented and discussed in “Prior VO Work” section. Finally, the conclusion for this article is presented in “Conclusions” section.

## Localization sensors and techniques

### Wheel odometry

**Fig. 2:**
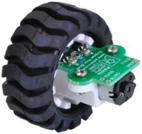
Wheel odometry with an optical encoder [Pololu Corporation 2016]

The simplest and most widely utilized method to estimate the position of mobile robots is wheel odometry. It is used to estimate wheeled vehicle position by counting the number of revolutions of the wheels that are in contact with the ground. Wheel revolutions can be translated accurately into linear displacement relative to the ground (Borenstein et al. 1996). Encoders are used to measure wheel rotation, as shown in Fig. 2.

Wheel odometry is a relative positioning technique. It suffers from position drift and inaccuracy because of wheel slippage, which leads to error accumulation over time (Fernandez and Price 2004; Nourani-Vatani et al. 2009). Translation and orientation errors in wheel odometry increase proportionally with the total travelled distance. Wheel odometry is simple and inexpensive, allows for high sampling rates, and exhibits good short-term accuracy (Borenstein et al. 1997; Aboelmagd et al. 2013).

### INS

**Fig. 3:**
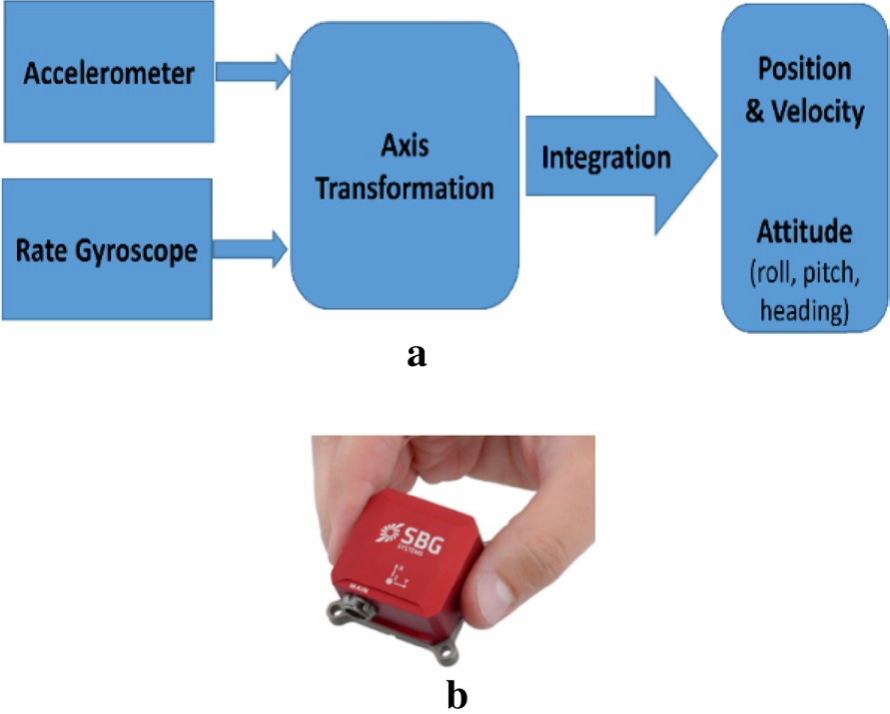
Inertial navigation system. **a** Block diagram of INS. **b** Miniature INS [SBG Systems 2016]

INS is a relative positioning technique that provides the position and orientation of an object relative to a known starting point, orientation, and velocity. As shown in Fig. 3, it is a navigation aid that uses a computer, motion sensors (accelerometers), and rotation sensors (rate gyroscopes) to continuously calculate the position, orientation, and velocity of a moving vehicle, which could be a ground vehicle, an airplane, a spaceship, a rocket, a surface ship, or a submarine. The advantage of INS is that it is self-contained, that is, it does not require external references (Wang et al. 2014; Woodman 2007).

However, INS is highly prone to drift accumulation because calculation of the change in velocity and position is implemented by performing successive mathematical integrations of acceleration with respect to time. Accelerometer data need to be integrated twice to yield the position, whereas rate-gyro data are only integrated once to track the orientation. Therefore, any small errors in the measurement of acceleration and angular velocity are integrated into large errors in velocity, which are compounded into still larger errors in position (Rone and Ben-Tzvi 2013; Wang et al. 2014; Woodman 2007). The errors are cumulative and increase with time. Thus, the position needs to be periodically corrected with the input of another navigation system.

Consequently, inertial sensors are inaccurate and unsuitable for positioning applications over an extended period of time and are usually utilized to supplement other navigation systems, such as GPS, to provide a higher degree of accuracy than is possible with the use of any single system (Maklouf and Adwaib 2014). Moreover, accurate inertial navigation requires high-cost equipment. Thus, the high cost of a highly precise INS makes the method an unviable solution for commercial purposes (Borenstein et al. 1996, 1997).

### GPS/GNSS

GNSS is used as an umbrella term for all current and future global radio-navigation systems including the U.S. GPS, the Russian global navigation satellite system (GLONASS), and the European Georgia Library Learning Online System (GALILEO). At present, there are two navigation satellite systems in orbit which are GPS and GLONASS. GALILEO is planned to be deployed and operational by 2013 (Rizos et al. 2010).

Before GPS was invented in the early 1970s by the U.S. Department of Defense (DoD), the primary method of navigation revolved around the map and compass. GPS is a satellite-based navigation system that allows users to accurately determine their location anywhere on or slightly above the surface of the Earth (El-Rabbany 2002; Cook 2011).

**Fig. 4:**
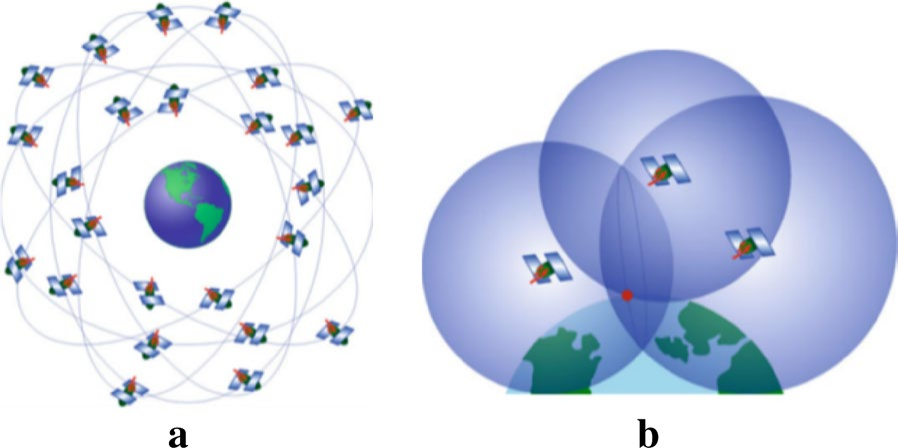
Global positioning system [Aboelmagd et al. 2013]. **a** GPS satellite constellation. **b** Concept of positioning by trilateration (*red dot* represents user’s position)

GPS is utilized for more than simple outdoor navigational exercises; it is used in geology, agriculture, landscaping, construction, and public transportation. GPS provides accurate position, navigation, and timing information free of charge to anyone who has a GPS receiver. GPS consists of a nominal constellation of 24 operational satellites orbiting the Earth and transmitting encoded radio frequency (RF) signals. They are arranged so that four satellites are placed in each of six orbital planes to ensure continuous worldwide coverage, as shown in Fig. 4a (El-Rabbany 2002; Aboelmagd et al. 2013).

Only four satellites are needed to provide positioning or location information. Through trilateration, ground receivers can calculate their position by using the travel time of the satellite’s signals and information about their current location that is included in the transmitted signal. Each satellite is equipped with a radio transmitter and receiver and atomic clocks. The receiver clocks are not as precise as the atomic clocks and normally exhibit bias. This bias generates errors in the travel time of the signals and leads to errors in the calculation of the distances to the satellites. Theoretically, by using the principle of trilateration/triangulation, a GPS receiver requires the ranges to three satellites only to calculate the 3D position (latitude, longitude, and altitude), but a fourth satellite is required to estimate the offset of the receiver’s clock from the system clock and to correct clock bias in the receiver. Figure 4b shows the concept of GPS positioning by trilateration using three satellites (Aboelmagd et al. 2013; Cook 2011).

GPS provides the absolute position with a known ratio of error. Its main advantages are its immunity to error accumulation over time and its long-term stability. GPS is a revolutionary technology for outdoor navigation; it is effective in areas with a clear view of the sky but is unusable for indoor, confined, underground, and underwater spaces. The limitations of GPS include outages caused by satellite signal blockage, occasional high noise content, multipath effects, low bandwidth, and interference or jamming. GPS outages occur in urban canyons, tunnels, and other GPS-denied environments and confined places (Gonzalez et al. 2012; Maklouf and Adwaib 2014; Cook 2011; Wang et al. 2014).

**Fig. 5:**
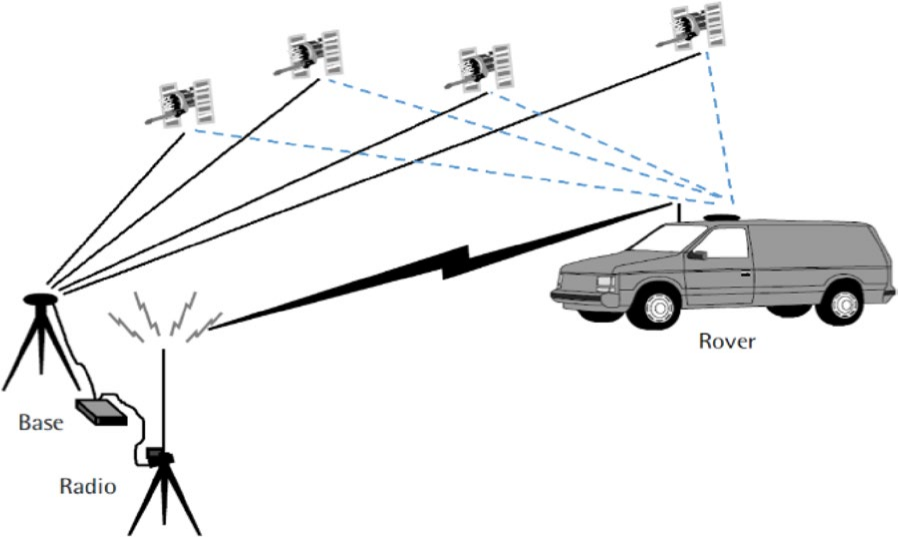
Real-time differential global positioning system

Common standalone GPS is used for positioning and has an accuracy within 10 m. Differential GPS (DGPS) and real-time kinematic GPS (RTK-GPS) were invented to improve GPS accuracy and allow for localization in outdoor open-field environments within a sub-meter or centimeter order. They are relative positioning techniques that employ two or more receivers simultaneously to track the same satellites. DGPS mainly consists of three elements: one GPS receiver (base station) located at a known location, one GPS receiver (user receiver) called a rover, and a radio communication medium between these two receivers (Fig. 5). DGPS can correct bias errors of the user receiver by using measured bias errors at the base station (Aboelmagd et al. 2013; Morales and Tsubouchi 2007).

RTK-GPS provides real-time measurements in centimeter accuracy. It provides two solutions, namely, float and fix. The first solution requires a minimum of four common satellites and provides an accuracy range of approximately 20 cm to 1 m. The second RTK-GPS solution requires at least five common satellites and provides accuracy within 2 cm (Aboelmagd et al. 2013; Cook 2011).

### Sonar/ultrasonic sensors

Sonar/ultrasonic sensors utilize acoustic energy to detect objects and measure distances from the sensor to the target objects. They have two main parts, namely, transmitter and receiver. The transmitter sends a short ultrasonic pulse, and the receiver receives what comes back of the signal after it has reflected off nearby objects. The sensor measures the time-of-flight (TOF), which is the time from signal transmission to reception. Given that the transmission rate of an ultrasonic signal is known, the distance to the target that reflects the signal can be computed. Sonar sensors can be utilized to localize mobile robots through model matching or triangulation by computing the pose change between every two sensor inputs acquired at two different poses. By combining many sonar sensors, a sonar array can obtain a detailed picture of the environment and exhibit high positioning accuracy (Jiménez and Seco 2005; Kreczmer 2010).

The major drawback of these sensors is the reflection of signal waves that are highly dependent on the material and the orientation of the object surface. Moreover, they are sensitive to noise from the environment and other robots using ultrasound with the same frequency. Many objects in the environment are assumed to be specular reflectors for ultrasonic waves, which cause a sonar sensor to receive a multi-reflected echo instead of the first one (Kreczmer 2010; Rone and Ben-Tzvi 2013; Sanchez et al. 2012).

### Laser sensors

Laser sensors can be utilized in several applications related to positioning. It is a remote sensing technology for distance measurement that involves transmitting a laser toward the target and then analyzing the reflected light. Laser-based range measurements depend on either TOF or phase-shift techniques. Similar to the sonar sensor, in a TOF system, a short laser pulse is sent out, and the time until it returns is measured. This type of sensor is often referred to as laser radar or light detection and ranging sensor (LIDAR). However, in phase-shift systems, a continuous signal is transmitted. The phase of the returned signal is compared with a reference signal generated by the same source. The velocity of the target and the distance to it are measured with the Doppler shift (Horn and Schmidt 1995; Takahashi 2007).

LIDAR is mostly used in obstacle detection and avoidance, mapping, and 3D motion capture. LIDAR can be integrated with GPS and INS to enhance the accuracy of outdoor positioning applications. Although sonar sensors have a large beam width that allows for increased coverage, the angular resolution with a laser scanner is much better than that with an ultrasonic one (Aboelmagd et al. 2013; Lingemann et al. 2005).

A drawback of LIDAR when compared with sonar sensors is that it entails a highly expensive solution. Moreover, analysis of LIDAR data has a high computational cost and may affect the response of real-time applications. The iterative manner of calculating the optimal match between two laser scans increases the computational cost. Furthermore, scanning can fail when the material appears as transparent for the laser, such as glass, because the reflections on these surfaces lead to suspicious data (Takahashi 2007; Horn and Schmidt 1995; Lingemann et al. 2005).

### Optical cameras

Cameras and vision systems can be employed in mobile robotic applications for localization and to perform various tasks. Recently, many researchers have been showing interest in visual-based localization systems because these systems are more robust and reliable than other sensor-based localization systems. Camera images can be utilized for indoor and outdoor vehicle navigation, such as to detect road edges, lanes, and their transitions as well as road intersections. The images captured by a camera can provide a large amount of information to be used for several purposes, including localization. Compared with proximity sensors, optical cameras are low-cost sensors that provide a large amount of meaningful information. Moreover, they are passive; that is, visual localization systems do not suffer from the interferences often encountered when active ultrasonic or laser proximity sensors are used (Frontoni 2012; Rone and Ben-Tzvi 2013).

Vision-based navigation of mobile robots is one of the main goals of computer vision and robotics research (Campbell et al. 2005). This approach is a non-contact method for the effective positioning of mobile robots, particularly in outdoor applications (Nagatani et al. 2010). For autonomous navigation, a robot needs to track its own position and motion. VO provides an incremental online estimation of the vehicle position by analyzing the image sequences captured by a camera (Campbell et al. 2005; Gonzalez et al. 2012). Vision-based odometry is an inexpensive alternative technique that is relatively more accurate than conventional techniques, such as GPS, INS, and wheel odometry (Howard 2008). VO has a good trade-off among cost, reliability, and implementation complexity (Nistér et al. 2004). It can estimate robot location inexpensively by using a consumer-grade camera instead of expensive sensors or systems, such as GPS and INS (Nistér et al. 2006; Nourani-Vatani et al. 2009).

However, image analysis is typically computationally expensive. In visual localization, the computations involve several steps, namely, (1) acquisition of camera images, (2) extraction of several image features (edges, corners, lines, etc.), (3) matching between image frames, and (4) calculation of the position by calculating the pixel displacement between frames. Moreover, vision algorithms are highly sensitive to operating and environmental conditions, such as lightning, textures, illumination changes throughout the day, presence of blurs in images, presence of shadows, and presence of water or snow on the ground. Therefore, these algorithms may perform well under several conditions, but in other environmental conditions, it will not work well and thus become unreliable (Aboelmagd et al. 2013).

Table 1 shows a summary of the features and drawbacks of the six most commonly used localization technologies.

**Table 1:** Comparison of commonly used localization sensors

Sensor/technology	Advantages	Disadvantages
Wheel odometry	Simple to determine position/orientation Short term accuracy, and allows high sampling rates Low cost solution	Position drift due to wheel slippage Error accumulation over time Velocity estimation requires numerical differentiation that produces additional noise
INS	Provides both position and orientation using 3-axis accelerometer and gyroscope Not subject to interference outages	Position drift (position estimation requires second-order integral) Have long-term drift errors
GPS/GNSS	Provides absolute position with known value of error No error accumulation over time	Unavailable in indoor, underwater, and closed areas Affected by RF interference
Ultrasonic sensor	Provides a scalar distance measurement from sensor to object Inexpensive solution	Reflection of signal wave is dependent on material or orientation of obstacle surface Suffer from interference if multiple sensors are used Low angular resolution and scan rate
Laser sensor	Similar to sonar sensors but has higher accuracy and scan rate Return the distance to a single point (rangefinder) or an array of distances (scanner)	Reflection of signal wave is dependent on material or orientation of obstacle surface Expensive solution
Optical camera	Images store a huge meaningful information Provide high localization accuracy Inexpensive solution	Requires image-processing and data-extraction techniques High computational-cost to process images

The process of estimating ego-motion (translation and orientation of an agent (e.g., vehicle, human, and robot)) by using only the input of a single or multiple cameras attached to it is called VO (Scaramuzza and Fraundorfer 2011).

## VO

Localization is the main task for autonomous vehicles to be able to track their paths and properly detect and avoid obstacles. Vision-based odometry is one of the robust techniques used for vehicle localization. This section comprehensively discusses VO and its types, approaches, applications, and challenges.

### What is VO?

VO is the pose estimation process of an agent (e.g., vehicle, human, and robot) that involves the use of only a stream of images acquired from a single or from multiple cameras attached to it (Scaramuzza and Fraundorfer 2011). The core of VO is camera pose estimation (Ni and Dellaert 2006). It is an ego-motion online estimation process from a video input (Munguia and Gra 2007). This approach is a non-contact method for the effective positioning of mobile robots (Nagatani et al. 2010). VO provides an incremental online estimation of a vehicle’s position by analyzing the image sequences captured by a camera (Campbell et al. 2005; Gonzalez et al. 2012).

The idea of estimating a vehicle’s pose from visual input alone was introduced and described by Moravec in the early 1980s (Nistér et al. 2004; Scaramuzza and Fraundorfer 2011). From 1980 to 2000, VO research was dominated by NASA in preparation for the 2004 Mars Mission. The term “visual odometry” was coined by Nistér et al. (2004). The term was selected because vision-based localization is similar to wheel odometry in that it incrementally estimates the motion of a vehicle by integrating the number of turns of its wheels over time (Scaramuzza and Fraundorfer 2011). In the same manner, VO integrates pixel displacements between image frames over time.

### Why VO?

VO is an inexpensive and alternative odometry technique that is more accurate than conventional techniques, such as GPS, INS, wheel odometry, and sonar localization systems, with a relative position error ranging from 0.1 to 2% (Scaramuzza and Fraundorfer 2011). This method is characterized by good balance among cost, reliability, and implementation complexity (Nistér et al. 2004). The use of a consumer-grade camera instead of expensive sensors or systems, such as GPS, INS, and laser-based localization systems, is a straightforward and inexpensive method to estimate robot location (Nistér et al. 2006; Gonzalez et al. 2012; Nourani-Vatani et al. 2009). Although GPS can be utilized for outdoor localization, lost GPS information causes significant errors (Takahashi 2007).

Images store large amounts of meaningful information, which are sufficient to estimate the movement of a camera (Rone and Ben-Tzvi 2013). VO is unaffected by wheel slippage in uneven terrains or other unfavorable conditions. Furthermore, VO works effectively in GPS-denied environments (Scaramuzza and Fraundorfer 2011). The rate of local drift under VO is smaller than the drift rate of wheel encoders and low-precision INS (Howard 2008). VO can be integrated with GPS and INS for maximum accuracy.

Different from laser and sonar localization systems, VO does not emit any detectable energy into the environment. Moreover, compared with GPS, VO does not require the existence of other signals (Ni and Dellaert 2006). Compared with the use of other sensors, the use of cameras for robot localization has the advantages of cost reduction, allowing for a simple integration of ego-motion data into other vision-based algorithms, such as obstacle, pedestrian and lane detection, and without the need for calibration between sensors (Wang et al. 2011). Cameras are small, cheap, lightweight, low powered, and versatile. Thus, they can also be employed in any vehicle (land, underwater, air) and for other robotic tasks (e.g., object detection and recognition).

### VO challenges

Although indoor robot localization has been implemented successfully, robot localization in outdoor environments remains a challenging problem. Many factors, (e.g., terrains are usually not flat, direct sunlight, shadows, and dynamic changes in the environment caused by wind and sunlight) make localization difficult in outdoor environments (Takahashi 2007). The main challenges in VO systems are mainly related to computational cost and light and imaging conditions (Gonzalez et al. 2013; Nagatani et al. 2010; Nourani-Vatani and Borges 2011; Yu et al. 2011).

For VO to work efficiently, sufficient illumination and a static scene with enough texture should be present in the environment to allow apparent motion to be extracted (Scaramuzza and Fraundorfer 2011). In areas that have a smooth and low-textured surface floor, directional sunlight and lighting conditions are highly considered, leading to non-uniform scene lighting. Moreover, shadows from static or dynamic objects or from the vehicle itself can disturb the calculation of pixel displacement and thus result in erroneous displacement estimation (Gonzalez et al. 2012; Nourani-Vatani and Borges 2011).

Monocular vision systems suffer from scale uncertainty (Kitt et al. 2011; Cumani 2011; Zhang et al. 2014). If the surface is uneven, the image scale will fluctuate, and the image scaling factor will be difficult to estimate. According to Kitt et al. (2011), estimation of the scaling factor may become erroneous when a large change in the road slope occurs, which may lead to incorrect estimation of the resulting trajectory.

### VO applications

VO has a wide range of applications and has been effectively applied in several fields. Its application domains include robotics, automotive, and wearable computing (Scaramuzza and Fraundorfer 2011; Fraundorfer and Scaramuzza 2012). VO is applied in many types of mobile robotic systems, such as ground, underwater, aerial, and space robots. In space exploration, for example, VO is used to estimate the ego-motion of the NASA Mars rovers (Maimone et al. 2007). NASA utilizes VO to track the motion of the rovers as a supplement to dead reckoning.

VO is mainly used for navigation and to reach targets efficiently as well as to avoid obstacles while driving. It is also applied in unmanned aerial vehicles (UAVs) to perform autonomous take-off and landing and point-to-point navigation. Moreover, VO plays a significant role in autonomous underwater vehicles and coral-reef inspection systems (Dunbabin et al. 2005). Given that the GPS signal degrades or becomes unavailable in underwater environments, underwater vehicles cannot rely on GPS for pose estimation; therefore, VO is considered a cost-effective solution for underwater localization systems.

In the automotive industry, VO also plays a big role. It is applied in numerous driver assistance systems, such as vision-based assisted braking systems. VO is considered a cost-effective solution compared with LIDAR systems (Fraundorfer and Scaramuzza 2012). In ground vehicle robotics, the effective use of visual sensors for navigation and obstacle detection is the main goal (Nistér et al. 2006). VO is employed in cases where the GPS signal is unavailable (in planetary environments), too heavy to carry (on a small air vehicle), or insufficiently accurate at a low cost (in agricultural applications) (Zhang et al. 2014; Jiang et al. 2014a). It is also used in agricultural field robots to estimate the robot’s position relative to the crops (Ericson and Astrand 2008; Jiang et al. 2014a).

### Types of camera used in VO

**Fig. 6:**
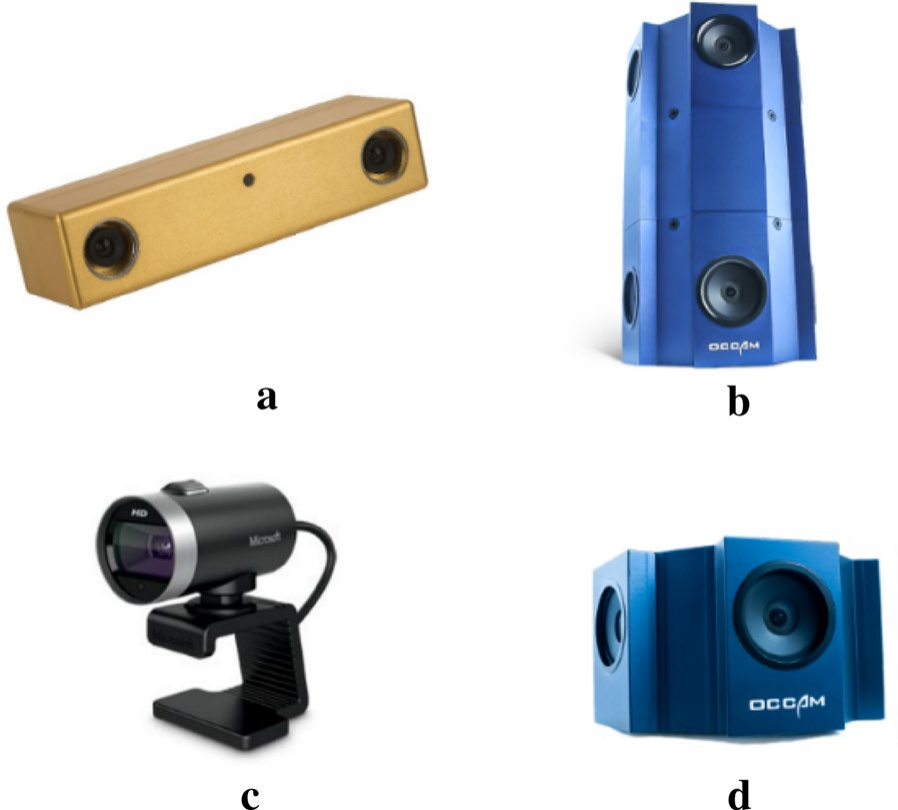
Different types of camera used in VO systems. **a** Stereo camera [courtesy of VOLTRIUM]. **b** Stereo omnidirectional [courtesy of Occam]. **c** Monocular camera [courtesy of Microsoft]. **d** Monocular omnidirectional [courtesy of Occam]

VO can be classified according to the type of camera/data sensor utilized to estimate the robot trajectory (Valiente García et al. 2012). Various types of camera, such as stereo, monocular, stereo or monocular omnidirectional, and RGB-D cameras (Fig. 6), can be used for VO purposes.

Most VO methods that have been proposed in existing literature use either stereo or monocular cameras and can be roughly classified as stereo or monocular VO systems. The systems that utilize a binocular camera are considered stereo VO systems, as implemented by Nistér et al. (2006), Howard (2008), Azartash et al. (2014), Golban et al. (2012), Soltani et al. (2012), Siddiqui and Khatibi (2014), Mouats et al. (2014), Alonso et al. (2012), McManus et al. (2013), Jiang et al. (2013), García-García et al. (2008), Martinez (2015); those that use a monocular camera are considered monocular VO systems, as applied by Yu et al. (2011), Gonzalez et al. (2012, 2013), Lovegrove et al. (2011), Martinez (2013), Nagatani et al. (2010), Nourani-Vatani et al. (2009), Royer et al. (2007), Jiang et al. (2014b).

A binocular camera has two lenses, with a separate image sensor for each lens. It has been used on Mars to estimate robot motion since early 2004 (Nistér et al. 2006). Given that information on the third dimension (i.e., depth) can be extracted from a single frame, the image scale can be immediately and instantaneously retrieved because the size of the stereo baseline is fixed and known, thereby resulting in an efficient and accurate triangulation process. Moreover, the various features present in both types of cameras increase the tracking ability in subsequent frames (Gonzalez et al. 2012; Nistér et al. 2006). However, stereo cameras are more expensive than conventional cameras. In addition, binocular cameras require more calibration effort than monocular cameras, and errors in calibration directly affect the motion estimation process (Kitt et al. 2011). Furthermore, it is very important for stereo VO that the two images of the stereo pair to be acquired at exactly the same time interval. That can be achieved by synchronizing the shutter speed of the two cameras of stereo vision or by synchronizing the two cameras by an external trigger signal provided by the controlling PC through serial or parallel port (Krešo et al. 2013; Cumani and Guiducci 2008). Much more effort is required to maintain a calibrated constant baseline between the pair of cameras than to maintain a single calibrated camera. Stereo VO can be degraded to the monocular case when the stereo baseline is much smaller than the distances to the scene from the camera. Stereo vision becomes ineffective in this case, and monocular methods are recommended (Scaramuzza and Fraundorfer 2011; Sünderhauf and Protzel 2007).

Using a monocular camera mitigates the effect of calibration errors in motion estimation. Low cost and easy deployment are the main motivations for using the monocular camera in many common applications, such as cellular phones and laptops. However, monocular vision systems suffer from scale uncertainty (Kitt et al. 2011; Cumani 2011). As discussed by Nagatani et al. (2010), Kitt et al. (2011), Nourani-Vatani et al. (2009), Gonzalez et al. (2012), Cumani (2011), if the surface is uneven, the image scale will fluctuate, and the image scaling factor will become difficult to estimate. According to (Kitt et al. 2011), estimation of the scaling factor may become erroneous if a large change in the road slope occurs, which may lead to incorrect estimation of the resulting trajectory. Monocular VO systems, compared with stereo VO systems, are essentially good for small robotics because they conserve the space of the baseline between the pair of stereo cameras. Moreover, interfacing and synchronization are more difficult with stereo cameras than with monocular cameras.

Several VO systems utilize omni-directional cameras, as presented by Scaramuzza and Siegwart (2008a), Valiente García et al. (2012), Bunschoten and Krose (2003), Scaramuzza and Siegwart (2008b), Scaramuzza et al. (2010), Tardif et al. (2008), and several others employ RGB-D cameras that provide both color and dense depth images, as presented by Fabian and Clayton (2014a), Steinbrücker et al. (2011), Huang et al. (2011), Fang and Zhang (2015), Fabian and Clayton (2014b), Dryanovski et al. (2013), Kerl et al. (2013), Whelan et al. (2013). Omni-directional cameras can represent a scene with a very wide field of vision (FOV) (up to 360° FOV). Given that omni-directional cameras can provide more information than common cameras and their features stay in the camera FOV for a longer period of time, a well refined 3D model of the world structure can be generated (Valiente García et al. 2012).

Table 2 shows a summary of the features and drawbacks of the three most commonly used cameras for VO. Each type of camera has advantages and disadvantages, so no single type can provide a 100% perfect solution.

**Table 2:** Comparison between types of cameras used for VO

Type of VO camera	Pros	Cons
Monocular	Low cost and easy deployment Light weight: good for small robotics Simple calibration	Suffer from image scale uncertainty
Stereo	Image scale and depth information is easy to be retrieved Provide 3D vision	More expensive and needs more calibration effort than monocular cameras It is degraded to the monocular case when the stereo baseline is much smaller than the distances to the scene from the camera Difficult interfacing and synchronization.
Omnidirectional	Provides very wide field of vision (FOV) (up to 360° FOV) Can generate well refined 3D model of the world structure Rotational invariance	Complex system Multiple cameras calibrating and synchronizing Needs high bandwidth Expensive

### Approaches of VO

Estimating the position of a mobile robot with vision-based odometry can generally be approached in three ways: through a feature-based approach, an appearance-based approach, or a hybrid of feature- and appearance-based approaches (Scaramuzza and Fraundorfer 2011; Valiente García et al. 2012).

#### Feature-based approach

The feature-based approach, as used by Nistér et al. (2006), Howard (2008), Cumani (2011), Benseddik et al. (2014), Naroditsky et al. (2012), Jiang et al. (2013), Villanueva-Escudero et al. (2014), Parra et al. (2010), involves extracting image features (such as corners, lines, and curves) between sequential image frames, matching or tracking the distinctive ones among the extracted features, and finally estimating the motion. In this approach, matching an image with a previous one is accomplished by comparing each feature in both images and calculating the Euclidean distance of feature vectors to find the candidate matching features. Afterward, the displacement is obtained by calculating the velocity vector between the identified pairs of points (Lowe 2004; Nistér et al. 2004, 2006). If stereo VO is implemented, the extracted features from the first frame are matched with the corresponding points in the second frame, thus providing the 3D position of the points in space. The camera motion is estimated based on feature displacement where relative pose of camera can be estimated by finding the geometric transformation between two images acquired by the camera using a set of corresponding feature points. To compute the matching between the feature points of two images, nearest neighbour pairs among their feature descriptors have to be determined. An 8-point algorithm was proposed by Longuet-Higgins to compute the pose via the essential matrix (Longuet-Higgins 1981). This method is similar to the structure from motion (SfM) method (Kicman and Narkiewicz 2013). Many works have been implemented to improve the robustness of Longuet-Higgins approach (Hartley 1997, Wu et al. 2005) or to solve it efficiently in a closed-form algorithm with the minimal set of five points (Nistér 2004). In (Nistér 2004), the relative camera pose was estimated from five matching feature points. However, several algorithms use 6, 7, and 8 feature pairs for relative motion estimation (Stewenius et al. 2006). Feature-based VO has been successfully utilized as the navigation system of Mars exploration rovers (Maimone et al. 2007) as well as in the missions of the Mars Science Laboratory (Johnson et al. 2008).

Kalman filter is one of the important Bayesian filters used to improve the accuracy and refine the VO estimation results (Van Hamme et al. 2015). It uses a prior vehicle state estimate to predict current feature locations and then compares this prediction to current observations to calculate an updated vehicle state. The state estimates delivered by the Kalman Filter utilizes any available information to minimize the mean of the squared error of the estimates with regard to the available information (Lin et al. 2013). In Helmick et al. (2004), a Kalman filter pose estimator has been implemented with VO system for autonomous rover in high slip environments. In this Helmick work, salient features in stereo images were tracked and a maximum likelihood motion estimation algorithm was used to estimate rover motion between successively acquired stereo image pairs. The Kalman filter merges data from an Inertial Measurement Unit (IMU) and VO. This merged estimate is then compared to the kinematic estimate to determine if and how much slippage has occurred. If slippage has occurred then a slip vector is calculated by differencing the current Kalman filter estimate from the kinematic estimate to be used for calculating the necessary wheel velocities and steering angles to compensate for slip and follow the desired path.

#### Appearance-based approach

The appearance-based approach, as implemented by Gonzalez et al. (2012, 2013), Lovegrove et al. (2011), Yu et al. (2011), Nourani-Vatani et al. (2009), Nourani-Vatani and Borges (2011), McManus et al. (2013), Zhang and Kleeman (2009), Bellotto et al. (2008), monitors the changes in the appearance of acquired images and the intensity of pixel information therein instead of extracting and tracking features. It focuses on the information extracted from the pixel intensity (Valiente García et al. 2012). The camera motion and vehicle speed can be estimated using optical flow (OF). OF algorithm uses the intensity values of the neighboring pixels to compute the displacement of brightness patterns from one image frame to another (Campbell et al. 2004; Barron et al. 1994). Algorithms that estimate the displacement for all the image pixels are known as dense OF algorithms such as the Horn-Schunck algorithm which calculates the displacement at each pixel by using global constraints (Horn and Schunck 1982). However, algorithms that calculate the displacement for a selected number of pixels in the image are called sparse optical flow algorithms such as the Lucas-Kanade method (Lucas and Kanade 1981). Dense algorithms avoid feature extraction but are less robust to noise compared to sparse OF algorithms. Therefore, sparse OF algorithms are desirable over dense OF algorithms for many VO applications (Campbell et al. 2005, Corke et al. 2004, Nourani-Vatani and Pradalier 2010). In sparse algorithms, the features should be chosen carefully, considering that pixels in regions with more variance between the neighbors will produce more reliable displacement estimation.

The commonly used method in appearance-based approach is the template matching method. The template matching method selects a patch or a template from the current image frame and attempts to match this patch in the next frame. Vehicle displacement and rotation angle are retrieved by matching a template between two consecutive image frames. Template matching is a main task in various computer vision applications. It is extensively applied in various areas, such as object detection, video compression, and automatic inspection (Yoo et al. 2014; Brunelli 2009). Template matching is the process of determining the existence and position of a sub-image or an object inside a larger scene image (Choi and Kim 2002; Goshtasby et al. 1984). The sub-image is called the template, and the larger image is called the search area. Template matching decides whether the template exists in the search area and determines its location if it does. It computes the degree of similarity between the template and search area by shifting the template over the search area and calculating the degree of similarity in each location based on various similarity measures. The shift position that has the largest similarity degree is the likely position of the template found in the search area (Yoo et al. 2014; Jurie and Dhome 2002; Goshtasby et al. 1984; Choi and Kim 2002).

The main similarity measures that are widely used in template matching are sum of squared or absolute differences (SSD/SAD) and normalized cross correlation (NCC) (Yoo et al. 2014; Goshtasby et al. 1984). NCC as a measure is more accurate than SSD/SAD. However, NCC algorithms are computationally slower than SSD/SAD algorithms (Goshtasby et al. 1984; Choi and Kim 2002; Yoo et al. 2014). Given that NCC-based template matching computes the normalized cross correlation of intensity values between two windows, it is considered one of the most common template matching methods that are invariant to linear brightness and contrast variations (Mahmood and Khan 2012; Zhao et al. 2006a, b).

**Fig. 7:**
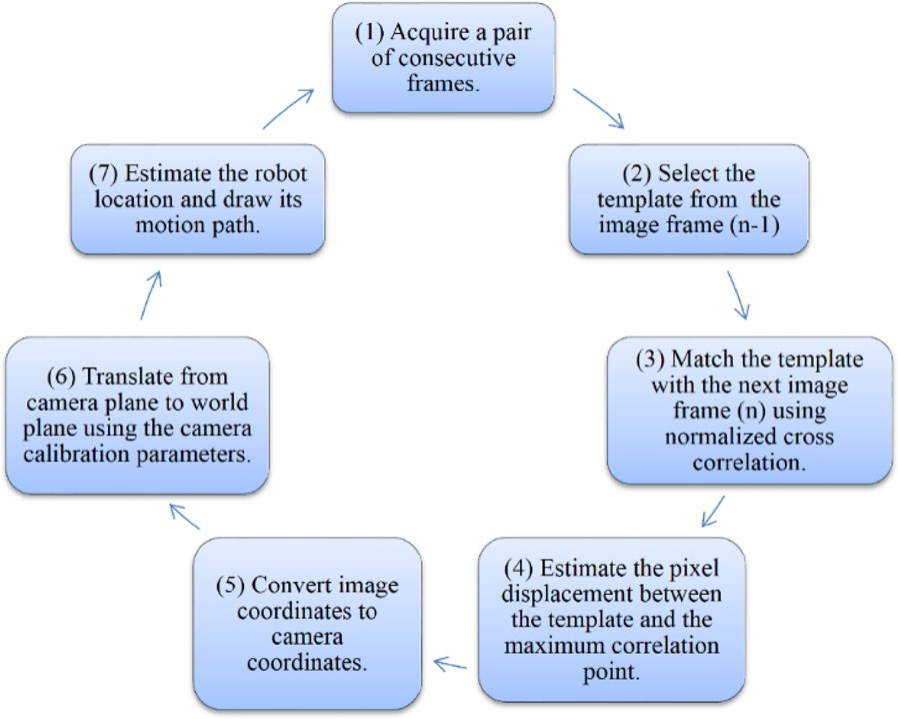
Flowchart of visual odometry system algorithm

Figure 7 presents the flowchart of the required steps of the VO system algorithm using correlation-based template matching (Aqel et al. 2016). The algorithm begins by acquiring a pair of consecutive image frames. Thereafter, the template is selected from the first frame and then matched with the next frame through normalized cross correlation. Then, the pixel displacement between the template and the maximum correlation point is calculated. Once the horizontal and vertical pixel displacements (Δ*u* and Δ*v*) are measured, these pixel displacements are converted to the physical horizontal and vertical camera displacement (in meters) by using the intrinsic and extrinsic camera calibration parameters through the following equations:

1 $$\begin{aligned} \Delta X_{c} = - \Delta u\left( {\frac{{Z_{c} }}{{f_{x} }}} \right) \hfill \\ \Delta Y_{c} = - \Delta v\left( {\frac{{Z_{c} }}{{f_{y} }}} \right) \hfill \\ \end{aligned}$$

The 2D image coordinate frame is converted to the camera coordinate frame by reversing the directions of the X and Y axes because image and camera coordinates are opposite each other.

For the camera coordinate plane (*X*_*c*_ *,Y*_*c*_ *,Z*_*c*_) to be converted to the vehicle coordinate plane (*X*_*v*_ *,Y*_*v*_ *,Z*_*v*_) with the application of Euler angles, rotation matrix *R*_*c*_ was calculated by rotating the camera coordinate plane 180° around the Z-axis and then by 180° around the new Y-axis, as depicted in Eqs. (2) and (3).

2 $$R_{c} = R_{z} \times R_{y} = \left[ {\begin{array}{*{20}c} {\cos (\theta_{z} )} & { - \sin (\theta_{z} )} & 0 \\ {\sin (\theta_{z} )} & {\cos (\theta_{z} )} & 0 \\ 0 & 0 & 1 \\ \end{array} } \right] \times \left[ {\begin{array}{*{20}c} {\cos (\theta_{y} )} & 0 & {\sin (\theta_{y} )} \\ 0 & 1 & 0 \\ { - \sin (\theta_{y} )} & 0 & {\cos (\theta_{y} )} \\ \end{array} } \right],$$

3 $$\left[ {\begin{array}{*{20}c} {\Delta X_{{v_{i} }} } \\ {\Delta Y_{{v_{i} }} } \\ {\Delta Z_{{v_{i} }} } \\ \end{array} } \right] = R_{c} \times \left[ {\begin{array}{*{20}c} {\Delta X_{{c_{i} }} } \\ {\Delta Y_{{c_{i} }} } \\ {\Delta Z_{{c_{i} }} } \\ \end{array} } \right].$$

As the motion model was assumed to be as an Ackerman-steered model, the physical vehicle displacement (translation Δ*X* and rotation Δ*θ*) in the vehicle coordinate plane is then calculated using Eq. (4).

4 $$\begin{aligned} \Delta X_{i} = \Delta X_{{v_{i} }} , \hfill \\ \Delta \theta_{i} = \tan^{ - 1} \left(\frac{{\Delta Y_{{v_{i} }} }}{{L_{cam} }}\right), \hfill \\ \end{aligned}$$

where Δ*X*_*v*_ and Δ*Y*_*v*_ are the vehicle displacement in the vehicle coordinate frame, and *L*_*cam*_ is the distance between the camera center and the vehicle’s center of rotation. Finally, the new position (*P*_*new*_) of the vehicle in the world coordinate plane is calculated using Eq. (5) which is equal to the previous position (*P*_*previous*_) plus the incremental translation (*T*_*incremental*_ *)* in the X-axis direction and, using the rotation matrix *R*_*Z*-*axis*_, rotated around the Z-axis by a heading angle equal to *θ*_i + 1_.

5 $$P_{new} = P_{previous} + R_{{Z - axis_{{}} }} \times T_{Incremental} ,$$

#### Hybrid of feature- and appearance-based approaches

The feature-based approach is suitable for textured scenarios, such as rough and urban environments (Johnson et al. 2008; Gonzalez et al. 2012). However, this approach fails to deal with texture-less or low-textured environments of a single pattern (e.g., sandy soil, asphalt, and concrete). The few salient features that can be detected and tracked in these low-textured environments make the feature-based approach inefficient in such environments (Nourani-Vatani et al. 2009; Nourani-Vatani and Borges 2011; Gonzalez et al. 2012; Johnson et al. 2008). By contrast, the appearance-based approach is more robust and superior to feature tracking methods in low-textured (Kicman and Narkiewicz 2013; Nourani-Vatani and Borges 2011). Given that a large template can be employed in the matching process with this method, the probability of successful matching between two consecutive image frames is high.

In some scenarios, hybrid approach is the best solution which is a combination of feature- and appearance- based approaches. They combine between tracking salient features over the frames and using the pixel intensity information of the whole or batch of image. For example, in Scaramuzza and Siegwart (2008a), the hybrid approach was implemented because the appearance-based approach alone was not very robust to image occlusions. Therefore, in their work, image features from the ground plane was used to estimate the vehicle translation while the image appearance was used to estimate the rotation of the vehicle.

## Prior VO work

Vision-based odometry can estimate robot location inexpensively by using a consumer-grade camera instead of expensive sensors or systems, such as GPS and INS (Nistér et al. 2006; Gonzalez et al. 2012; Nourani-Vatani et al. 2009). It provides an incremental online estimation of the vehicle position by analyzing the image sequences captured by a camera (Campbell et al. 2005; Gonzalez et al. 2012). VO as an effective non-contact positioning method, particularly in outdoor applications, is one of the main goals in computer vision and robotics research (Campbell et al. 2005; Nagatani et al. 2010). It is characterized by good trade-off among cost, reliability, and implementation complexity (Nistér et al. 2004).

### Camera attachment to vehicle

In existing literature, most VO systems have cameras mounted and attached to the vehicle, either oriented toward the ground or faced forward. A downward-facing camera was utilized by Nourani-Vatani et al. (2009), Yu et al. (2011), Nourani-Vatani and Borges (2011), Lovegrove et al. (2011), Nagatani et al. (2010), Kadir et al. (2015), Zienkiewicz and Davison (2014) for vehicle position estimation with an appearance-based template matching approach. Two monocular cameras were used by Gonzalez et al. (2012): a downward-facing monocular camera for displacement and a front-facing camera as a visual compass to estimate the vehicle orientation. Although the forward-facing camera provides more information than the downward-facing camera, template matching or feature tracking with the forward-facing camera can be disturbed by shadows and dynamic changes in the environment caused by wind and sunlight (Piyathilaka and Munasinghe 2010; Dille et al. 2010). Moreover, a forward-facing VO system under low-light conditions requires the surrounding environment to be illuminated and possibly requires more power than the vehicle can provide.

### Stereo VO

Estimating a vehicle’s ego-motion by using only visual inputs was introduced in the early 1980s by Moravec (1980). Most of the early VO research was driven by NASA to develop a VO system for planetary rovers with the capability to estimate motion in Mars, which has uneven and rough terrains. Moravec used a planetary rover equipped with a single camera sliding on a rail, which is called a slider stereo. The rover moved in a stop-and-go manner. In each stop location, the camera slid horizontally and captured nine images at equidistant intervals. By using his proposed corner detector, the corners were detected in an image and matched through NCC. Finally, motion was estimated by triangulation of the 3D points seen at two consecutive robot positions. Although Moravec utilized a single sliding camera, his work is related to the class of stereo VO algorithms.

Matthies and Shafer (1987) utilized a stereo system and Moravec’s approach to detect and track corners; he obtained good results with 2% relative error on a 5.5 m trajectory for a planetary rover. Nistér et al. (2004) coined the term “visual odometry” and demonstrated the first real-time long-run implementation with a robust outlier rejection scheme. They did not use Moravec’s approach to track features among stereo frames, but they detected features independently in all frames and only allowed matches between features. This scenario avoids feature drift during cross correlation-based tracking. In Cheng et al. (2005), the importance of stereo VO during NASA’s missions with the rovers Spirit and Opportunity was presented. Other recent work on stereo VO for different types of robots in different environments were presented by Nistér et al. (2006), Howard (2008), Azartash et al. (2014), Golban et al. (2012), Soltani et al. (2012), Li et al. (2013). A real-time stereo VO system was implemented by Howard (2008) for ground vehicles through feature matching rather than tracking and employing stereo range data for inlier detection.

Stereo VO was implemented by Helmick et al. (2004) to allow a Mars rover to accurately follow paths in high-slip environments and to estimate its travelling motion. It depends on tracking distinctive scene features in stereo imagery and estimates the change in the position and altitude of two or more pairs of stereo images by using maximum likelihood motion estimation. A correlation-based search and tracking based on an affine template was implemented to precisely determine the 2D positions of selected features in the second image pair and to eliminate the tracking error caused by a large roll and scale change between images. Stereo matching was then performed on these tracked features in the second pair to determine their new 3D positions. The slippage rate was computed with the Kalman filter, which merges the estimates from VO and IMU and compares the estimates with the motion estimate from vehicle kinematics.

### Monocular VO

When the distance to the scene from the stereo camera is much larger than the stereo baseline, stereo VO can be degraded to the monocular case, and stereo vision becomes ineffectual (Scaramuzza and Fraundorfer 2011, Sünderhauf and Protzel 2007). In monocular VO, both the relative motion and 3D structure are computed from 2D bearing data (Scaramuzza and Fraundorfer 2011). Successful works that employed VO with a single camera have been conducted in the last decade by using both monocular (Yu et al. 2011; Gonzalez et al. 2012, 2013; Lovegrove et al. 2011; Martinez 2013; Nagatani et al. 2010, Nourani-Vatani et al. 2009; Van Hamme et al. 2015; Lee et al. 2015; Forster et al. 2014; Villanueva-Escudero et al. 2014) and omnidirectional cameras (Yu et al. 2011; Gonzalez et al. 2012; 2013; Lovegrove et al. 2011; Martinez 2013; Nagatani et al. 2010; Nourani-Vatani et al. 2009; Scaramuzza and Siegwart 2008a; Corke et al. 2004; Bunschoten and Krose 2003; Valiente García et al. 2012).

In Nistér et al. (2004), a real-time VO that can estimate motion from a monocular or stereo camera has been developed. Furthermore, the first real-time large-scale VO with a monocular camera was presented. It uses feature tracking approach and random sample consensus (RANSAC) for outlier rejection. The new upcoming camera pose was computed through 3D to 2D camera-pose estimation. The developed algorithm, which consists of three phases (feature detection, feature tracking, and motion estimation), can be applied to either monocular or stereo vision systems, with a slight change in the motion estimation phase. The algorithm begins by extracting corners from each image frame and then tracking the detected features between frames. A matching criterion is implemented to successfully track features from one image to the next. Finally, the motion estimation phase is executed. In the case of a monocular vision system, the motion estimation phase calculates the pose for each tracked feature by using a five-point pose algorithm. Afterward, the 3D position of each detected feature is calculated with the first and last acquired images. Next, 3D point information is used for the estimation of the 3D pose of the camera. In a stereo vision system, the 3D position of each extracted feature is calculated through stereo matching of the features between the two images obtained by each of the cameras.

Van Hamme et al. proposed a monocular VO algorithm which uses planar tracking of feature points on the world ground plane surrounding the vehicle rather than traditional 3D pose estimation (Van Hamme et al. 2015). For easy consistency of motion among features, feature tracking was applied not in the image coordinates of the perspective camera but in the ground plane coordinates. An online self-learning approach of monocular VO and ground classification for ground vehicles were presented by Lee et al. (2015). A constrained kinematic model was utilized to solve the motion and structure problem and to estimate the ground surface. A probabilistic appearance-based ground classifier that is learned online was used for effective sampling in the geometric search for the ground points. Thus, a combination of geometric estimates with appearance-based classification was performed to achieve an online self-learning scheme from monocular vision. Forster et al. presented a semi-direct monocular VO algorithm that is applied to a micro aerial vehicle (Forster et al. 2014). This algorithm operates directly on pixel intensities and eliminates the need for feature extraction and matching techniques in motion estimation. It uses a probabilistic mapping method that explicitly models outlier measurements to estimate 3D points.

A monocular omnidirectional VO using a hybrid combination of feature- and appearance-based approaches was developed by Scaramuzza and Siegwart (2008a). The features from the ground plane were used by tracking scale-invariant feature transform (SIFT) points to estimate the translation and absolute scale. An image appearance visual compass was used to estimate the rotation of the vehicle. The feature-based approach was also utilized to detect failures of the appearance-based method because it is not robust to obstructions.

In Piyathilaka and Munasinghe (2010), an experimental study on the use of VO for short-run self-localization of field robots was presented. Fast Fourier transform (FFT) based on image registration techniques was applied to calculate the relative translation and orientation between consecutive frames captured from a ground surface by a downward-facing monocular camera. The results of this study showed that FFT fails when the ground surface is low-textured and has repeated features, such as cut grass, gravel, and sand.

Simultaneous localization and mapping (SLAM) is a technique allows robots to operate in an environment without a priori knowledge of a map (Souici et al. 2013). By SLAM, robot can localize itself in an unknown environment and incrementally generate a map of this environment while at the same time using this map to estimate its new pose relative to this map. Visual SLAM use camera sensors to acquire observation data to be used in building the map. In features-based SLAM, SLAM use environment to update the position of the robot by extracting features from the environment and re-observing when the robot moves around. For example, LSD-SLAM and ORB-SLAM are real-time algorithm for simultaneous localization and mapping with a monocular freely-moving camera (Engel et al. 2014; Mur-Artal et al. 2015). ORB-SLAM is a feature-based approach robust to severe motion clutter, allows wide baseline loop closing and re-localization, and includes full automatic initialization. ORB features have enough recognition power to enable place recognition from severe viewpoint change and very fast to extract and match (without the need of multithreading acceleration) that enable real-time accurate tracking and mapping. However, LSD-SLAM uses direct approach which does not need feature extraction and thus avoid the corresponding artefacts. It is able to generate semi-dense reconstructions of the environment, while the camera is localized by optimizing directly over image pixel intensities. Moreover, it is robust to blur, low-texture environments like asphalt.

### VO limitations

According to Gonzalez et al. (2013), Nagatani et al. (2010), Nourani-Vatani and Borges (2011), Yu et al. (2011), the main limitations of VO systems are related to the computational cost and light and imaging conditions (i.e., direct sunlight, shadows, image blur, and image scale variance). In areas that have a smooth and low-textured surface floor, the directional sunlight and lighting conditions are highly considered, which leads to non-uniform scene lighting. Moreover, the shadows from static or dynamic objects and from the vehicle itself can disturb the calculation of pixel displacement, which causes errors in displacement estimation (Gonzalez et al. 2012, Nourani-Vatani and Borges 2011).

In Gonzalez et al. (2012, 2013), Yu et al. (2011), Nourani-Vatani et al. (2009), Nourani-Vatani and Borges (2011), Siddiqui and Khatibi (2014), a monocular VO was implemented through NCC template matching for ground car-like vehicles. In these studies, the best positioning accuracy was achieved with less than 3% error of the total travelling distance. The limitations of these systems are related to the negative effects of shadows, image blur, and deficiency in dealing with scale variance at uneven surfaces. These limitations lead to false matching, which increases the estimation errors.

### Scale uncertainty

According to Kitt et al. (2011), Cumani (2011), Choi et al. (2015), monocular vision systems are negatively affected and may fail because of scale uncertainty. In stereo VO systems, the scale of motion can be recovered by using the baseline between the two cameras as a reference. However, in monocular VO systems, scale ambiguity is unsolvable when camera motion is unconstrained (Zhang et al. 2014). As discussed by Nagatani et al. (2010), Kitt et al. (2011), Nourani-Vatani et al. (2009), Gonzalez et al. (2012), Cumani (2011), estimating the fluctuated image scale factor on an uneven terrain is difficult. According to Kitt et al. (2011), when a large change in road slope occurs, estimation of the scaling factor may become erroneous, which may lead to incorrect estimation of the resulting trajectory. The relative scale with respect to the previous frames is determined using either knowledge of the 3D structure or the trifocal tensor because the absolute image scale is unknown. Therefore, the absolute scale can be determined from direct measurements (e.g., measuring the size of an object in the scene), motion constraints, or integration with other sensors, such as inertial measurement unit (IMU) and range sensors (Scaramuzza and Fraundorfer 2011; Hartley and Zisserman 2004). Scale ambiguity can be overcome by using independent information on the observed scene, such as the actual size of known objects (Cumani 2011). As discussed by (Nagatani et al. 2010, Nourani-Vatani et al. 2009), image scale variance occurs when a robot moves on non-smooth or loose soil floors that make the wheels go up or down; then, the distance between the camera and the ground changes, and the image zooms in and out. This image scale fluctuation affects the images (makes them shorter and wider than the actual scene), prevents correct matching for visual tracking, and results in poor and unreliable motion estimation. Several sensors, such as laser range finder, acceleration, and IMU sensors, can be utilized to measure the camera height fluctuation (Gonzalez et al. 2012).

Recovering the image scale is possible when the camera motion is constrained to a surface. For example, in Kitt et al. (2011), image scale ambiguity was solved by using the Ackermann steering model and assuming that the vehicle drives on a planar road surface. In Nourani-Vatani and Borges (2011), the planar motion of a vehicle was estimated by using a downward-facing camera and the Ackermann steering model for estimation. Moreover, an INS system is used to obtain vehicle pitch and roll angles. To resolve the image scale variation problem, (Nourani-Vatani and Borges 2011; Gonzalez et al. 2012) regarded the distance between the downward-facing camera and the ground as almost constant because the differences in camera height were cancelled throughout the experiment as zero mean. In Scaramuzza et al. (2009), a monocular camera positioned with an offset to the vehicle rotation center was used to recover scale as the vehicle turns. However, the formulation degenerates in straight driving, and the scale is no longer recoverable. In Zhang et al. (2014), a method that does not require the imaged terrain to be flat was demonstrated. The method can simultaneously recover the inclination angle of the ground and estimate the motion. Wheel odometry deals with cases in which the detected terrain is not flat. Recently, a new approach was designed and applied by Nagatani et al. (2010). The author developed a telecentric camera by using a CCD camera and telecentric lens that maintains the same FOV regardless of the variation in camera height from the ground.

In Guo and Meng (2012), a system for VO and obstacle detection that involves the use of only a single camera was proposed. The Kanade–Lucas–Tomasi (KLT) feature tracker was utilized for feature extraction, and the RANSAC algorithm was used for outlier rejection. The relative pose between two consecutive frames was extracted from the essential matrix through SVD decomposition. Given that the absolute scale of the translation cannot be derived from monocular motion estimation, the scale ambiguity problem was solved by using the constraints of camera mounting and ground planar assumption. To detect obstacles and separate the ground and obstacle areas from each other, the image was segmented into regions. Each region was classified as either ground or off-ground according to three criteria: homography constraint, feature point distribution, and boundary point reconstruction.

Table 3 is a summary table of some VO prior works which illustrated in “Prior VO work” section.

**Table 3:** Summary table of some VO works in literature

Reference	Camera type	Approach	VO estimation accuracy	Limitations
Gonzalez et al. (2013)	Two monocular cameras: downward-facing camera for displacement and front-facing camera for orientation estimation	Appearance-based approach (NCC template matching)	Error <3% of the total travelling distance and <8° average orientation error	False matches due to shadows and blur at velocity >1.5 m/s Can’t deal with scale variance on non-smooth surfaces
Van Hamme et al. (2015)	Monocular camera	Feature-based approach (inverse perspective projection and Kalman filter for Tracking of features in the ground plane)	>8.5% translation error (for 800 m)	Significant rotational bias on some estimated trajectory segments due to non-planarity of the road environment in those segments
Scaramuzza and Siegwart (2008a)	Omnidirectional camera	Hybrid approach (tracking SIFT feature points from ground plane to estimate translation. Image appearance similarity measure (NCC, Manhattan and Euclidean distance) was used to estimate the rotation of the car)	Error is <2% of the distance traversed 5° average orientation error	Unavoidable visual odometry drift and deviation due to road humps that violate the planar motion assumption
Nistér et al. (2004)	Stereo camera	Feature-based approach (Detection of features independently in all frames and only allowed matches between salient features)	1.63% error over 380 m of the distance traversed	No mention
Howard (2008)	Stereo camera	Feature-based approach (Feature matching and employing stereo range data for inlier detection)	0.25% error over 400 m of the distance traversed	Self-Shadow leads to false-matches It does not work effectively on vegetated environment
Nourani-Vatani and Borges (2011)	Monocular camera	Appearance-based approach (NCC multi-template matching which selects best template based on entropy)	Error <5% of total travelling distance 5° average heading error	Deficiency in dealing with scale variance at uneven surfaces System can’t deal with sunny/shadow regions
Yu et al. (2011)	Monocular camera	Appearance-based approach (NCC rotated template matching)	1.38% distance error and 2.8° heading error	Cannot deal with image scale variance, shadows and blur
Nagatani et al. (2010)	Telecentric camera (which maintains the same field of ground area view, regardless of variation in camera height from ground	Appearance-based approach (cross correlation template matching)	<3% error indoor experiment 1.5% (for 100 m trajectory) at 0.4 m/s speed	Cannot estimate the camera height from ground variations
Zhang et al. (2014)	Monocular camera	Feature-based approach [tracking of features using Lucas Kanade Tomasi (LKT)]	Error is <1% of the distance traversed	Image scale uncertainty at complicated ground conditions for example loose soil floors

## Conclusions

VO and its types, approaches, and challenges were presented and discussed. The most common positioning sensors and techniques were presented, and their features and limitations were discussed and compared. Different sensors and techniques, such as wheel odometry, GPS, INS, sonar and laser sensors, and visual sensors, can be utilized for localization tasks. Each technique has its own drawbacks. VO is the localization of a robot using only a stream of images acquired from a camera attached to the robot. VO is a highly accurate solution to estimate the ego-motion of robots; it can avoid most of the drawbacks of other sensors. VO is an inexpensive solution and is unaffected by wheel slippage in uneven terrains.

Although GPS is the most common solution to localization because it can determine the absolute position without error accumulation, it is only effective in areas with a clear view of the sky. It cannot be used indoors and in confined spaces. The commercial GPS estimates position with errors in the order of meters. These errors are considered too large for precise applications that require accuracy in centimeters, such as autonomous parking. Differential GPS and real-time kinematic GPS can determine the position with centimeter accuracy, but these techniques are expensive. Meanwhile, VO works effectively in GPS-denied environments.

INS is highly prone to accumulating drift, and a highly precise INS is an expensive and unviable solution for commercial purposes. The rate of local drift under VO is smaller than the drift rate of wheel encoders and low-precision INS.

Generally, estimating the position of a mobile robot using the vision-based odometry technique can be approached in three ways: through a feature-based approach, an appearance-based approach, or a hybrid of feature- and appearance-based approaches. The feature-based approach is suitable for textured scenarios. Template matching method is highly appropriate for low-textured scenarios and is superior to feature tracking methods because it works robustly on almost texture-less surfaces.

The main challenges in VO systems are related to computational cost and light and imaging conditions (i.e., directional sunlight, shadows, image blur, and image scale/rotation variance). Most of the VO systems proposed in existing literature fail or cannot work effectively in outdoor environments with shadows and directional sunlight. Shadows and directional sunlight have negative effects that disturb the estimation of pixel displacement between image frames and lead to errors in vehicle position estimation.
